# Phenolic Compounds from *Belamcanda chinensis* Seeds

**DOI:** 10.3390/molecules23030580

**Published:** 2018-03-05

**Authors:** Ying-Ying Song, Ying Liu, Yong-Ming Yan, Xi-Feng Lu, Yong-Xian Cheng

**Affiliations:** 1 State Key Laboratory of Phytochemistry and Plant Resources in West China, Kunming Institute of Botany, Chinese Academy of Sciences, Kunming 650201, China; songyingying@mail.kib.ac.cn; 2 Guangdong Key Laboratory for Genome Stability & Disease Prevention, School of Pharmaceutical Sciences, Shenzhen University Health Science Center, Shenzhen 518060, China; cdzyb083@sina.com (Y.L.); yanym@szu.edu.cn (Y.-M.Y.); 3 University of Chinese Academy of Sciences, Beijing 100049, China; 4 College of Pharmacy, Henan University of Chinese Medicine, Zhengzhou 450008, China

**Keywords:** *Belamcanda chinensis*, belamcanosides A and B, GC analysis, HMGCR, SQLE, LDLR, SORT1

## Abstract

Two new sucrose derivatives, namely, belamcanosides A (**1**) and B (**2**), together with five other known compounds (**3**−**7**), were isolated from the seeds of *Belamcanda chinensis* (L.) DC. Their structures were identified based on spectroscopic data. Especially, the absolute configurations of fructose and glucose residues in **1** and **2** were assigned by acid hydrolysis, followed by derivatization and gas chromatography (GC) analysis. Among the known compounds, (−)-hopeaphenol (**3**), (+)-syringaresinol (**4**), and quercetin (**5**), were isolated from *B. chinensis* for the first time. In addition, biological evaluation of **1** and **2** against cholesterol synthesis and metabolism at the gene level was carried out. The results showed that compounds **1** and **2** could regulate the expression of cholesterol synthesis and metabolism-associated genes, including 3-hydroxy-3-methylglutaryl-coenzyme A reductase (HMGCR), squalene epoxidase (SQLE), low density lipoprotein receptor (LDLR), and sortilin (SORT1) genes in HepG2 cells.

## 1. Introduction

**Figure 1:**
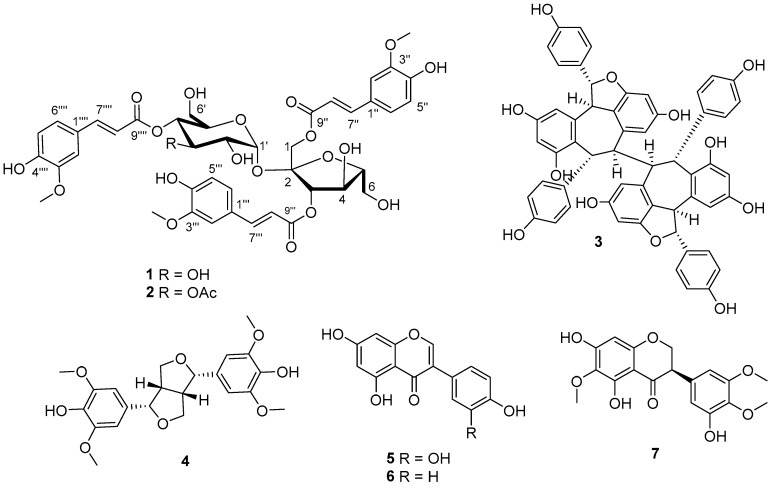
Chemical structures of compounds **1**–**7**.

*Belamcanda chinensis* (L.) DC., is an ornamental plant in the Iridaceae family. The dried rhizomes of *B. chinensis* have been used for the treatment of sore throat and pharyngitis in traditional Chinese medicine [1]. Benzoquinones, flavonoids, isoflavonoids, phenols, iridal-type triterpenoids, and steroids have been characterized from *B. chinensis* [2,3,4]. Among them, flavonoids and isoflavonoids were reported to be the main compounds, which showed a wide range of pharmacological activities such as antibacterial, anti-inflammatory, and antioxidant activity [5]. We have identified a potent Smad3 phosphorylation inhibitor, namely GQ5, from the dried resin of *Toxicodendron vernicifluum* [6] and renopreotctive substance linghzifuran A from *Ganoderma lucidum* [7]. The hybrids of GQ5 and lingzhifuran A, like belamcandones A and B, previously from the seeds of *B. chinensis* [8]. Therefore, *B. chinensis* seeds were investigated during our search for renoprotective compounds from natural origins. However, our efforts resulted in the isolation of two new sucrose derivatives, together with five known compounds (**3**–**7**) (Figure 1), as well as several structurally challengeable and unclarified belamcandone A analogues. This paper deals with the isolation, structural elucidation, and biological activity of these compounds.

## 2. Results and Discussion

### 2.1. Structure Elucidation of the Compounds

The acetone extract of *B. chinensis* was suspended in water and partitioned with EtOAc. The EtOAc extract was concentrated and submitted to a combination of chromatography to afford compounds **1**−**7**.

**Figure 2:**
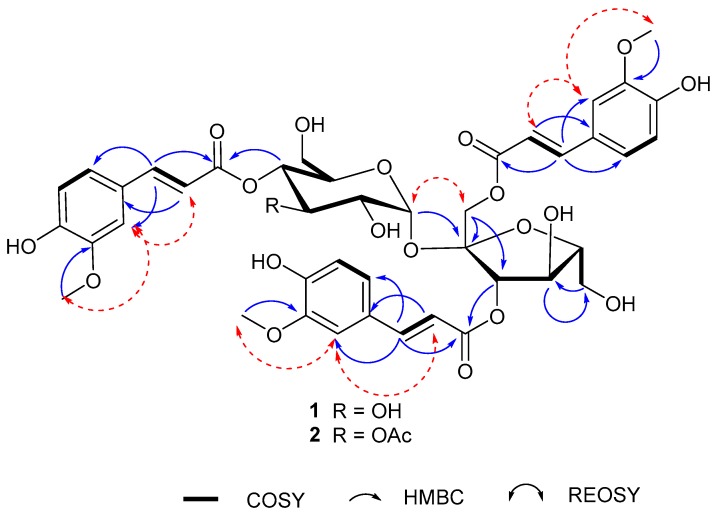
Key ^1^H-^1^H COSY, HMBC, and ROESY correlations of **1** and **2**.

Belamcanoside A (**1**), isolated as a light yellowish gum, has the molecular formula C_42_H_46_O_20_, based on analysis of its HRESIMS *m*/*z* 893.2478 [M + Na]^+^ (C_42_H_46_O_20_Na, calcd. for 893.2475) (supplementary materials), ^13^C-NMR and DEPT spectra, indicating 20 degrees of unsaturation. The ^1^H-NMR spectrum of **1** (Table 1) exhibits characteristic signals attributable to three pairs of olefinic protons [*δ*_H_ 7.76 (d, *J* = 15.9 Hz, H-7′′), 6.46 (d, *J* = 15.9 Hz, H-8′′), 7.57 (d, *J* = 15.9 Hz, H-7′′′), 6.19 (d, *J* = 15.9 Hz, H-8′′′), and 7.67 (d, *J* = 15.9 Hz, H-7′′′′), 6.38 (d, *J* = 15.9 Hz, H-8′′′′)], and three benzyl moieties with an ABX spin system [*δ*_H_ 7.15 (d, *J* = 1.6 Hz, H-2′′), 7.10 (dd, *J* = 8.2, 1.6 Hz, H-6′′), 6.75 (d, *J* = 8.2 Hz, H-5′′), 7.05 (d, *J* = 1.6 Hz, H-2′′′), 6.87 (dd, *J* = 8.2, 1.6 Hz, H-6′′′), 6.80 (d, *J* = 8.2 Hz, H-5′′′), 7.15 (d, *J* = 1.6 Hz, H-2′′′′), 7.06 (dd, *J* = 8.2, 1.6 Hz, H-6′′′′), and 6.78 (d, *J* = 8.2 Hz, H-5′′′′)], as well as three methoxyl groups at *δ*_H_ 3.80 (3H, s), 3.83 (3H, s), 3.84 (3H, s), respectively. The ^13^C-NMR and DEPT spectra of **1** indicate that it is comprised of 42 carbons ascribed to three methyls, 3 methylenes, 23 methines, and 13 quaternary carbons. These data indicate that **1** might contain three cinnamic acid residues. The ^1^H-^1^H COSY correlations of H-7′′/H-8′′, H-7′′’/H-8′′’, H-7′′′′/H-8′′′′, HMBC correlations of H-7′′, H-8′′/C-1′′, H-7′′’, H-8′′’/C-1′′’, H-7′′′′′, H-8′′′′/C-1′′′′, and ROESY correlations of three methoxyl groups respectively with H-2′′, H-2′′′, and H-2′′′′, secure the presence of three feruloyl moieties. The large coupling constants (15.9 Hz) of three pairs of olefinic protons suggested the trans-form of *α*,*β*-unsaturated carbonyl systems. Apart from three feruloyl residues, the ^1^H- and ^13^C-NMR spectra also show an α-anomeric resonance at *δ*_H_ 5.60, with a small coupling constant (d, *J* = 3.6 Hz, H-1′) due to a gauche conformation, according to the Karplus relation [9], together with the occurrence of a total twelve oxygenated carbon signals at *δ*_C_ 62.4–103.5. These data imply that **1** is a disaccharide containing both a pentose and a hexose moiety. On the basis of chemical evidence and detailed analysis of ^1^H-^1^H COSY, HSQC, and HMBC experiments (Figure 2), as well as comparison of spectroscopic data with its analogues [10,11,12,13], allowed to deduce that **1** is a phenylpropanoid sucroside. Consequently, compound **1** was concluded to be a sucrose derivative acylated by three feruloyl moieties. Upon inspection of the HMBC spectrum, correlations between H-1 (*δ*_H_ 4.37)/C-9′′ (*δ*_C_ 168.5) and H-3 (*δ*_H_ 5.57)/C-9′′′ (*δ*_C_ 168.2) in the fructofuranosyl moiety, and between H-4′ (*δ*_H_ 4.93)/C-9′′′′ (*δ*_C_ 168.5) in the glucopyanosyl, are observed, thus, the positions of the acyl residues were determined as shown (Figure 1). For the configuration of sugar moiety, acid hydrolysis of **1**, followed by TLC comparison and GC analysis, allows the assignment of d-fructose and d-glucose. In detail, the l-cysteine methyl ester hydrochloride derivatives of the hydrolysis products of **1**, d-,l-fructose and d-,l-glucose were prepared and subjected to GC comparison. The retention time for that of **1** is 19.909 min, 21.443 min, respectively (Figure S5), close to that of d-fructose (19.896 min) (Figure S1), d-glucose (21.393 min) (Figure S3), rather than l-fructose (19.449 min) (Figure S4), l-glucose (21.836 min) (Figure S5). Accordingly, the structure of **1** was assigned as (1,3-*O*-diferuloyl)-*β*-d-fructofuranosyl-(2→1)-(4-*O*-feruloyl)-*α*-d-glucopyranoside, and named belamcanoside A.

Belamcanoside B (**2**), was obtained as a light yellowish gum, and has the molecular formula C_44_H_48_O_21_ (21 degrees of unsaturation), based on analysis of its HRESIMS *m*/*z* 935.2585 [M + Na]^+^ ( C_44_H_48_O_21_Na calcd. for 935.2580) (Figure S14). The ^1^H- and ^13^C-NMR spectra of **1** and **2** (Table 1, Figure S16 and S17) are similar, except for additional signals for an acetyl group [*δ*_H_ 1.98 (3H, s, OAc-3′), *δ*_C_ 172.3 and *δ*_C_ 20.9 (OAc-3′)] in **2**. Moreover, the carbon signals for C-3′ (*δ*_C_ 74.5) and C-4′ (*δ*_C_ 70.2) in **2** are shifted downfield and upfield, respectively, relative to those in **1**. These data, together with a long range correlation between H-3' and the ester carbonyl group in the HMBC spectrum (Figure S20), imply that the acetyl group is present at the C-3′ of the glucopyranosyl moiety in **2**. Evidence for the presence of a d-fructose and a d-glucose residue in the structure of **2** comes from analysis of the acid hydrolysis products in the manner as described for **1**. Thus, the structure of **2** was determined to be that shown in Figure 1. Consequently, the structure of **2** was elucidated as (1,3-*O*-diferuloyl)-*β*-d-fructofuranosyl-(2→1)-(3-acetyl-4-*O*-feruloyl)-*α*-d-glucopyraoside, and named belamcanoside B.

Five known compounds were identified as (−)-hopeaphenol (**3**) [14], (+)-syringaresinol (**4**) [15], quercetin (**5**) [16], genistein (**6**) [17], and 2,3-dihydroirigenin (**7**) [18], respectively, by comparison their spectroscopic data with the literature data.

### 2.2. Cell Viability Assay

**Figure 3:**
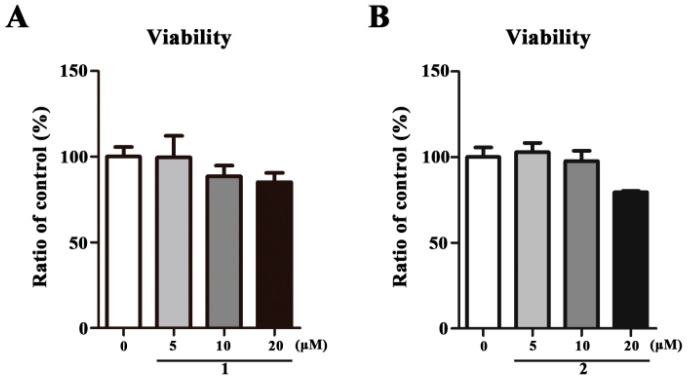
The effect of **1** and **2** on cell viability in HepG2 cells. HepG2 cells were treated with the indicated concentrations of **1** or **2** for 48 h followed by MTT assay. All the data are expressed as mean ± SEM (*n* = 3) and analyzed by ANOVA with the Bonferroni’s Test.

To exclude the biological activities that resulted from cytotoxicity, the cytotoxic effect of **1** and **2** at different concentrations (5, 10, and 20 μM) was firstly evaluated in HepG2 cells using MTT assay. We found that treatment with **1** or **2** for 48 h has no influence on cell viability (Figure 3A,B).

### 2.3. Effects on HMGCR and SQLE mRNA Expression

**Figure 4:**
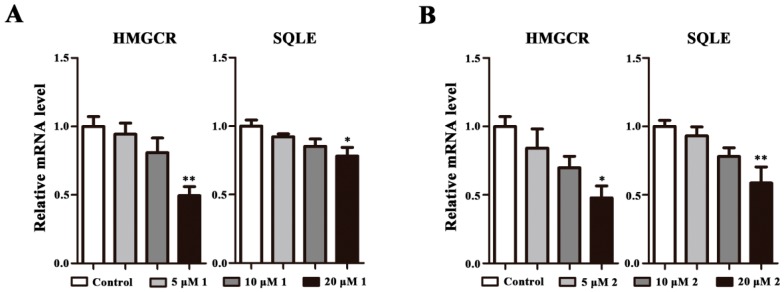
**1** and **2** stimulate the expression of HMGCR and SQLE genes. HepG2 cells were treated with **1** or **2** at 5, 10, and 20 μM for 48 h. Target mRNA levels are expressed relative to those in control by qPCR methods. All data are expressed as mean ± SEM (*n* = 3) and analyzed by ANOVA with Bonferroni’s test. * *p* < 0.05, ** *p* < 0.01 vs control.

It is known that HMGCR and SQLE are the two key control enzymes of cholesterol synthesis [19], we therefore investigated whether **1** or **2** could stimulate the function of HMGCR and SQLE. It was found that HMGCR and SQLE mRNA expression could be remarkably decreased by **1** or **2** at 20 μM (Figure 4A,B). Besides, these observations indicate that **1** and **2** could downregulate HMGCR and SQLE mRNA levels in a concentration-dependent manner.

### 2.4. Effects on LDLR and SORT1 mRNA Expression

**Figure 5:**
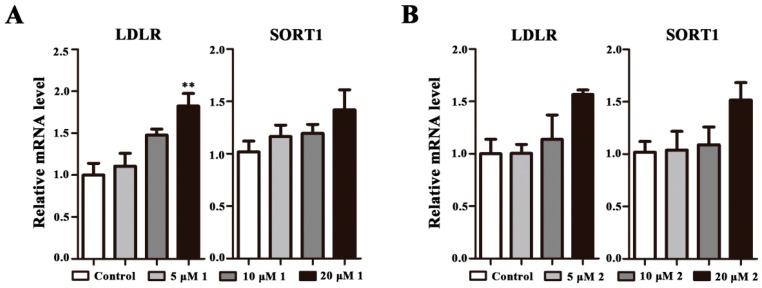
**1** or **2** stimulates the expression of LDLR and SORT1 genes. HepG2 cells were treated with **1** or **2** at 5, 10, and 20 μM for 48 h. Target mRNA levels are expressed relative to those in control by qPCR method. All data are expressed as mean ± SEM (*n* = 3), and analyzed by ANOVA with Bonferroni’s test. ** *p* < 0.01 vs control.

LDLR and SORT1 genes have recently been identified as novel regulators of cholesterol metabolism. Therefore, we investigated the two control points stimulated by **1** and **2**. The results show that **1** could increase LDLR mRNA expression significantly at 20 μM, but slightly increase SORT1 mRNA expression (Figure 5A). Additionally, the LDLR and SORT1 mRNA expression is increased, but there is no significant difference between the control and **2** (Figure 5B).

### 2.5. Discussion

Lipid metabolism is a complex physiological process that is involved in nutrient adjustment, hormone regulation, and homeostasis. An unhealthy lifestyle and chronic nutrient overload can cause lipid metabolism disorders, which may lead to serious lipid-related diseases, including obesity, non-alcoholic fatty liver disease (NAFLD), and type 2 diabetes mellitus (T2DM) [20]. Cells mainly regulate the balance among cholesterol synthesis, absorption, esterification, and outflow, to maintain normal cholesterol concentration. It has been reported that phenolic compounds play a role in suppressing hyperlipidaemia [21], decreasing fatty acid, cholesterol, triglycerides syntheses [22], and modulating lipogenic enzyme activities, such as ACC, FAS, DGAT, and HMGCR [23]. HMGCR and SQLE, that respectively catalyze the first and second rate-limiting steps in the cholesterol synthesis pathway, are genes involved in hepatic cholesterol biosynthesis [24,25]. HMGCR combines three molecules of acetyl-CoA with NADPH to synthesize mevalonate, the precursor of cholesterol biosynthesis, and then several steps of phosphorylation generate activated isoprenes, later condensed to form squalene, and cyclization of squalene occurs in several steps that finally generate the four fused rings characteristic of cholesterol and other sterols [26]. In our study, the phenolic compounds, two new sucrose derivatives isolated from *B. chinensis*, evidently decreased the HMGCR and SQLE mRNA expression in a dose-dependent correlation, implying their potential on improving cholesterol synthesis. Furthermore, belamcanoside A evidently increased the LDLR mRNA expression, which is a cell surface glycoprotein that plays a critical role in the homeostatic control of blood cholesterol by mediating the removal of cholesterol-containing lipoprotein particles from circulation [27], demonstrating an effect of belamcanoside A on cholesterol metabolism. However, both belamcanosides A and B have no obvious influence on SORT1 mRNA expression, which is a transmembrane type I trafficking receptor with a putative role in lipoprotein metabolism that plays an important role in reducing plasma cholesterol and triglycerides [28]. In summary, the two new compounds participate in the regulation of the key genes of lipid metabolism, revealing a potential benefit on cholesterol circular pathway. Therefore, the compounds are worty of in-depth study for the pharmacodynamics and molecular mechanism in vivo and in vitro.

## 3. Experimental Section

### 3.1. General Procedures

Column chromatography was carried out on silica gel (200–300 mesh; Qingdao Marine Chemical Inc., Qingdao, China), MCI gel CHP 20P (75–150 μm, Mitsubishi Chemical Industries, Tokyo, Japan), and Sephadex LH-20 (Amersham Pharmacia, Uppsala, Sweden). Optical rotations were recorded on a Jasco P-1020 polarimeter (Jasco Corporation, Tokyo, Japan). UV spectra were measured on a Shimadzu UV-2401PC spectrometer (Shimadzu Corporation, Tokyo, Japan). GC analysis was performed using an Agilent 6890N gas chromatography instrument (Agilent Technologies, Santa Clara, CA, USA). Semi-preparative or HPLC was carried out using an Agilent 1200 liquid chromatograph (Agilent Technologies, Santa Clara, CA, USA), the column used was a 250 mm × 9.4 mm, i.d., 5 µm. NMR spectra were recorded on an AV-400 (Bruker, Karlsruhe, Germany) or an AV-600 (Bruker, Karlsruhe, Germany) spectrometer with TMS as an internal standard. ESIMS and HRESIMS were collected by an UPLC-IT-TOF spectrometer (Shimadzu Corporation, Tokyo, Japan).

### 3.2. Plant Material

The dried seeds of *B. chinensis* were purchased from Shuyang, Jiangsu province, P.R. China, in August 2016, and were identified by Prof. Hua Peng, Laboratory for Plant Diversity and Biogeography of East Asia, Kunming Institute of Botany, Chinese Academy of Sciences, P.R. China. A voucher specimen (CHYX0603) has been deposited at the State Key Laboratory of Phytochemistry and Plant Resources in West China, Kunming Institute of Botany, Chinese Academy of Sciences, P.R. China.

### 3.3. Extraction and Isolation

The dried seeds of *B. chinensis* (20.1 kg) were powdered and extracted with acetone (4 × 50 L × 24 h) at room temperature, followed by concentration under reduced pressure to give an acetone extract (4.0 kg), which was suspended in H_2_O and partitioned with EtOAc three times to afford an EtOAc extract (2.2 kg). This extract was subjected to a MCI gel CHP 20P column with gradient aqueous MeOH (40–100%) to get ten fractions (Fr.A–Fr.J). Fr.E (27.4 g) was subjected to MCI gel CHP 20P with gradient aqueous MeOH (40%–100%) to give two subfractions (Fr.E1–Fr.E2). Fr.E2 (20.3 g) was subjected to Sephadex LH-20 column chromatography (MeOH) to give seven subfractions (Fr.E2.1–Fr.E2.7). Fr.E2.5 (134 mg) was subjected to Sephadex LH-20 (MeOH) and further purified by semi-preparative HPLC (MeCN/H_2_O, 26%, flow rate: 3 mL/min) to get compound **4** (1.5 mg, R_t_ = 29.8 min). Fr.E2.2 (608 mg) was subjected to preparative TLC (CHCl_3_/MeOH = 5:1) to give five subfractions (Fr.E2.2.1–Fr.E2.2.5). Fr.E2.2.3 (58 mg) was further purified by semi-preparative HPLC (MeCN/H_2_O, 35%, flow rate: 3 mL/min) to get compound **2** (5.5 mg, R_t_ = 27.8 min). Fr.E2.2.4 (67 mg) was further separated by semi-preparative HPLC (MeCN/H_2_O, 31%, flow rate: 3 mL/min) to get compound **7** (8.4 mg, R_t_ = 31.0 min). Fr.E2.2.4 (78 mg) was subjected to semi-preparative HPLC (MeCN/H_2_O, 27%, flow rate: 3 mL/min) to get compound **1** (6.3 mg, R_t_ = 19.0 min). Likewise, Fr.E2.5 (138 mg) was submitted to semi-preparative HPLC (MeCN/H_2_O, 35%, flow rate: 3 mL/min) to afford compounds **5** (2.1 mg, R_t_ = 13.8 min), **6** (2.8 mg, R_t_ = 29.1 min), and **3** (3.2 mg, R_t_ = 29.3 min).

### 3.4. Compound Characterization Data

*Belamcanoside A* (**1**): light yellowish gum; ${\left[\alpha \right]}_{D}^{21.9}$ +33.8 (*c* 0.74, MeOH); UV (MeOH) *λ*_max_ (log*ε*) 195 (4.38), 218 (4.49), 235 (4.43), 302 (4.51), 326 (4.65) nm; CD (MeOH) ∆*ε* (nm) + 5.8 (345); ESIMS *m*/*z* 893 [M + Na]^+^, HRESIMS *m*/*z* 893.2478 [M + Na]^+^ (calcd. for C_42_H_46_O_20_Na, 893.2475); ^1^H- and ^13^C-NMR data, see Table 1.

*Belamcanoside B* (**2**): light yellowish gum; ${\left[\alpha \right]}_{D}^{24.3}$ +19.6 (*c* 0.80, MeOH); UV (MeOH) *λ*_max_ (log*ε*) 195 (4.13), 218 (4.27), 235 (4.21), 302 (4.26), 326 (4.41) nm; CD (MeOH) ∆*ε* (nm) + 2.8 (346); ESIMS *m*/*z* 935 [M + Na]^+^, HRESIMS *m*/*z* 935.2585 [M + Na]^+^ (calcd. for C_44_H_48_O_21_Na, 935.2580); ^1^H- and ^13^C-NMR data, see Table 1.

### 3.5. Acid Hydrolysis and Sugar Analysis

A solution of **1** or **2** (**1**, 1 mg; **2**, 0.5 mg) in 1 N HCl was stirred at 70 °C for 5 h. After cooling, the mixtures were extracted with EtOAc. The aqueous layer was neutralized with 1 N NaOH and concentrated in vacuo, which was subsequently dissolved in anhydrous pyridine (2 mL). To these solutions, l-cysteine methyl ester hydrochloride (2.0 mg) was added, and the mixtures were stirred at 60 °C for 2 h, followed by concentrating in vacuo at 0 °C. Slow addition of 1-(trimethylsiyl) imidazole to the mixtures was followed by stirring at 60 °C for 1 h. Aliquots (2 µL) of the supernatants were subjected to chiral GC analysis to determine that d-fructose and d-glucose units present in **1** and **2** [24].

### 3.6. Cell Culture

Human liver carcinoma cells HepG2 were maintained in Dulbecco’s modifed Eagle’s medium (DMEM) supplemented with 10% (*v*/*v*) foetal bovine serum (FBS) and incubated at 37 °C with 5% CO_2_ atmosphere.

### 3.7. Cell Viability Assay

Cell viability was determined by MTT assay. HepG2 cells were plated in 96-well plates at a density of 5.0 × 10^3^ cells/well, and were cultured with medium for 24 h. After incubation for 48 h in the absence or presence of 5, 10, and 20 μM belamcanoside A or B respectively, a total of 10 µL of 0.5 mg/mL MTT solution was added into each well, and the cultures were further incubated for 4 h under the same condition. After removing the medium, DMSO (100 µL) was added to dissolve the formazan crystals. The samples were detected at 490 nm by an enzyme-labeled microplate reader.

### 3.8. RNA Isolation and qPCR

Total RNA was extracted from HepG2 cells using Direct-zol^TM^ RNA MiniPrep kit (ZYMO RESEARCH) according to the manufacturer’s instructions. cDNA was synthesized and amplifed from 1 μg of total RNA using the PrimeScript^TM^ RT Master Mix (TaKaRa), as described in the manufacturer’s protocol. qPCR analyses were performed with SYBR® Premix Ex Taq™ II (TaKaRa) under the following conditions: 30 s at 95 °C, 40 cycles at 95 °C for 5 s and 60 °C for 35 s. Gene expression levels were determined and normalized to the expression level of 36B4. The sequences of the primer sets used were h36B4, 5′-TCTAC AACCCTGAAGTGCTTGAT-3′ and 5′-CAATCTGCAGACAGACACTGG; hHMGCR, 5′-GAA TGGCCCTAGAACTGTGC-3′ and 5′-CAAAGAGCCCTGTGTGAATG-3′; hSQLE, 5′-CCTGA ATCAGAAAATAAGGAGCA-3′ and 5′-GCTTGTTTCTGAAATATTGGTTCC-3′; hLDLR, 5′-G CTTGTCTGTCACCTGCAAAT-3′ and 5′-AACTGCCGAGAGATGCACTT-3′; hSORT1, 5′-GG CATCATTGTGGCCATT-3′ and 5′-TTGACCTTCGTCTGTGGAGA-3′.

## 4. Conclusions

To conclude, this study led to the isolation of two new sucrose derivatives and three compounds firstly isolated from *B. chinensis*. Biological determination of the two new sucrose derivatives reveals that belamcanosides A and B at 20 μM could significantly reduce HMGCR and SQLE mRNA expression in HepG2 cells. Moreover, belamcanoside A could also promote LDLR mRNA expression in HepG2 cells at 20 μM. Both of them show good potency in regulating the cholesterol synthesis pathway, and exhibit low cytotoxicity. The present investigation adds new facets for the chemical composition of the seeds of *B. chinensis* and provides a foundation for further research of the two new sucrose derivatives.

## Figures and Tables

**Table 1:** ^1^H- (400 MHz) and ^13^C-NMR (150 MHz) data of **1** and **2** in CD_3_OD (*δ* in ppm, *J* in Hz).

	1			2	
Position	*δ*_H_ (*J*/Hz)	*δ*_C_ Mult	Position	*δ*_H_ (*J*/Hz)	*δ*_C_ Mult
1	4.37, s	66.7, t	1	4.39, s	66.5, t
2		103.5, s	2		103.5, s
3	5.57, d, (8.3)	79.9, d	3	5.62, d, (8.3)	79.9, d
4	4.46, t, (8.3)	73.3, d	4	4.44, t, (8.3)	73.5, d
5	3.98, m	84.2, d	5	4.00, m	84.3, d
6	3.85, m	63.0, t	6	3.86, m	63.1, t
1’	5.60, d, (3.6)	93.3, s	1’	5.66, d, (3.5)	92.2, s
2’	3.55, dd, (9.8, 3.6)	73.1, d	2’	3.74, m	71.1, d
3’	3.89, m	72.8, d	3’	5.47, t, (9.8)	74.5, d
4’	4.93, m	72.7, d	4’	5.12, t, (9.8)	70.2, d
5’	4.21, m	72.6, d	5’	4.30, brd (4.3)	72.4, d
6′	3.60, m	62.4, t	6′ OAc-3’	3.72, m	61.9, t
	3.71, brd, (12.1)			1.98, s	172.3, s
					20.9, q
1’’		127.5, s	1’’		127.6, s
2’’	7.15, d, (1.6)	111.6, d	2’’	7.11, d, (1.5)	111.7, d
3’’		149.3, s	3’’		149.2, s
4’’		150.7, s	4’’		150.8, s
5’’	6.75, d, (8.2)	116.4, d	5’’	6.72, d, (8.2)	116.3, d
6’’	7.10, dd, (8.2, 1.6)	124.5, d	6’’	7.03, dd, (8.2, 1.5)	124.4, d
7’’	7.76, d, (15.9)	114.8, d	7’’	7.65, d, (15.9)	114.9, d
8’’	6.46, d, (15.9)	148.0, d	8’’	6.35, d, (15.9)	147.7, d
9’’		168.5, s	9’’		168.5, s
OCH_3_	3.80, s	56.4, s	OCH_3_	3.80, s	56.3, s
1’’’		127.5, s	1’’’		127.6, s
2’’’	7.05, d, (1.6)	111.7, d	2’’’	7.03, d, (1.5)	111.6, d
3’’’		149.3, s	3’’’		149.3, s
4’’’		150.7, s	4’’’		150.6, s
5’’’	6.80, d, (8.2)	116.6, d	5’’’	6.70, d, (8.2)	116.4, d
6’’’	6.87, dd, (8.2, 1.6)	124.0, d	6’’’	7.11, dd, (8.2, 1.5)	124.6, d
7’’’	7.57, d, (15.9)	115.0, d	7’’’	7.74, d, (15.9)	115.0, d
8’’’	6.19, d, (15.9)	147.5, d	8’’’	6.45, d, (15.9)	148.1, d
9’’’		168.2, s	9’’’		168.2, s
OCH_3_	3.83, s	56.4, s	OCH_3_	3.76, s	56.4, s
1’’’’		127.6, s	1’’’’		127.2, s
2’’’’	7.15, d, (1.6)	111.7, d	2’’’’	7.15, d, (1.5)	111.8, d
3’’’’		149.4, s	3’’’’		149.2, s
4’’’’		151.0, s	4’’’’		150.9, s
5’’’’	6.78, d, (8.2)	116.6, d	5’’’’	6.73, d, (8.2)	116.6, d
6’’’’	7.06, dd, (8.2, 1.6)	124.0, d	6’’’’	7.03, dd, (8.2, 1.5)	124.3, d
7’’’’	7.67, d, (15.9)	114.9, d	7’’’’	7.53, d, (15.9)	114.2 , d
8’’’’	6.38, d, (15.9)	147.6, d	8’’’’	6.12, d, (15.9)	148.0, d
9’’’’		168.5, s	9’’’’		167.8, s
OCH_3_	3.84, s	56.4, s	OCH_3_	3.78, s	56.4, s

## Supplementary Materials

Supplementary materials are available online.
