# Supplementary material for: Phenolic Compounds from Belamcanda chinensis Seeds
Source: Molecules. 2018 Mar 5;23(3):580. doi: 10.3390/molecules23030580 (PMC6017503; doi:10.3390/molecules23030580)
Supplement: Supplementary file 1 [file molecules-23-00580-s001.pdf]

Supplementary Information

Phenolic compounds from *Belamcanda chinensis* seeds

Ying-Ying Song[1,2], Ying Liu[3], Yong-Ming Yan[3], Xi-Feng Lu[3]* and Yong-Xian Cheng[3,4]*

[1] *State Key Laboratory of Phytochemistry and Plant Resources in West China, Kunming Institute of Botany, Chinese Academy of Sciences, Kunming 650201, China; songyingying@mail.kib.ac.cn*

[2] *University of Chinese Academy of Sciences, Beijing 100049, China*

[3] *Guangdong Key Laboratory for Genome Stability & Disease Prevention, School of Pharmaceutical Sciences, Shenzhen University Health Science Center, Shenzhen 518060, China; cdzyb083@sina.com (Y.L.); yanym@szu.edu.cn (Y.-M.Y.)*

[4] *College of Pharmacy, Henan University of Chinese Medicine, Zhengzhou 450008, China*

* Correspondence: x.lu@szu.edu.cn (X.-F.L.); yxcheng@szu.edu.cn (Y.-X.C.); Tel./Fax: (+86) 0755-8671-3994

Content

Figure S1. GC analysis of the derivative of D-fructose
Figure S2. GC analysis of the derivative of L- fructose
Figure S3. GC analysis of the derivativ of D-glucose
Figure S4. GC analysis of the derivative of L-glucose
Figure S5. GC analysis of the derivative of compound **1** after hydrolysis
Figure S6. GC analysis of the derivative of compound **2** after hydrolysis
Figure S7. The ^{1}H NMR spectrum of **1** in CD_3OD
Figure S8. The ^{13}C NMR and DEPT spectra of **1** in CD_3OD
Figure S9. ^{1}H-^{1}H COSY spectrum of **1** in CD_3OD
Figure S10. HSQC spectrum of **1** in CD_3OD
Figure S11. HMBC spectrum of **1** in CD_3OD
Figure S12. ROESY spectrum of **1** in CD_3OD
Figure S13. CD spectrum of **1**
Figure S14. UV spectrum of **1**
Figure S15. HRESIMS of **1**
Figure S16. The ^{1}H NMR spectrum of **2** in CD_3OD
Figure S17. The ^{13}C NMR and DEPT spectra of **2** in CD_3OD
Figure S18. ^{1}H-^{1}H COSY spectrum of **2**
Figure S19. HSQC spectrum of **2**
Figure S20. HMBC spectrum of **2**
Figure S21. ROESY spectrum of **2**
Figure S22. CD spectrum of **2**
Figure S23. UV spectrum of **2**
Figure S24. HRESIMS of **2**

1. Supplementary figures

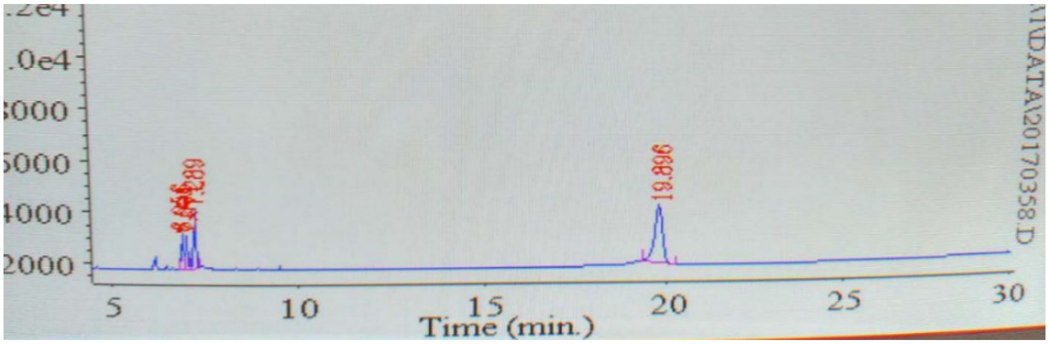

Figure S1. GC analysis of the derivative of D-fructose

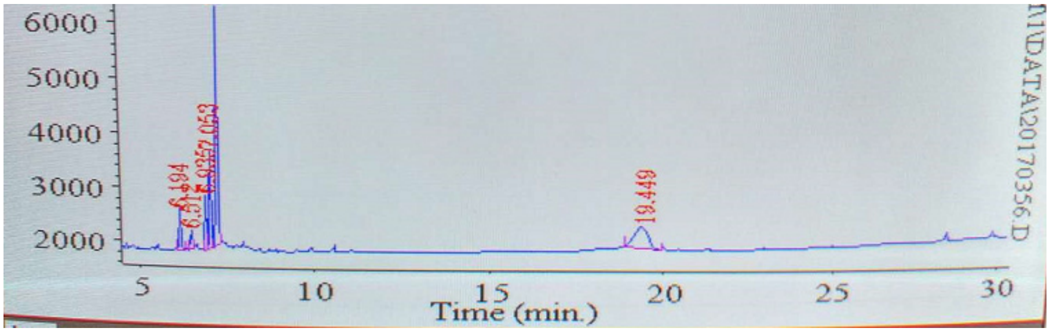

Figure S2. GC analysis of the derivative of L- fructose

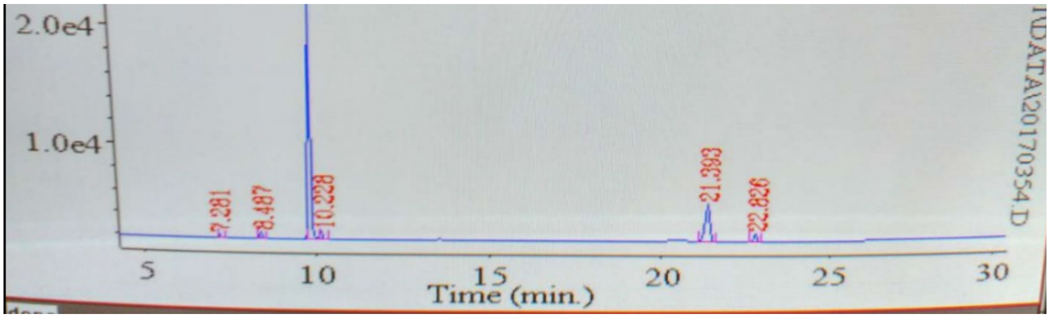

Figure S3. GC analysis of the derivative of D-glucose

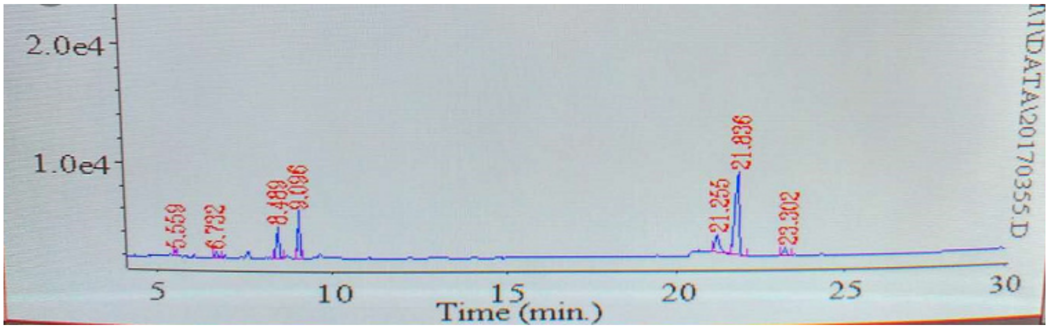

Figure S4. GC analysis of the derivative of L-glucose

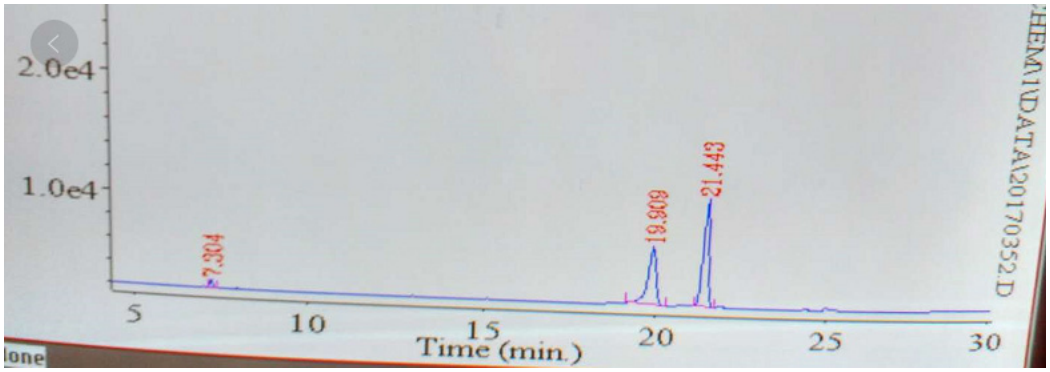

Figure S5. GC analysis of the derivative of compound **1** after hydrolysis

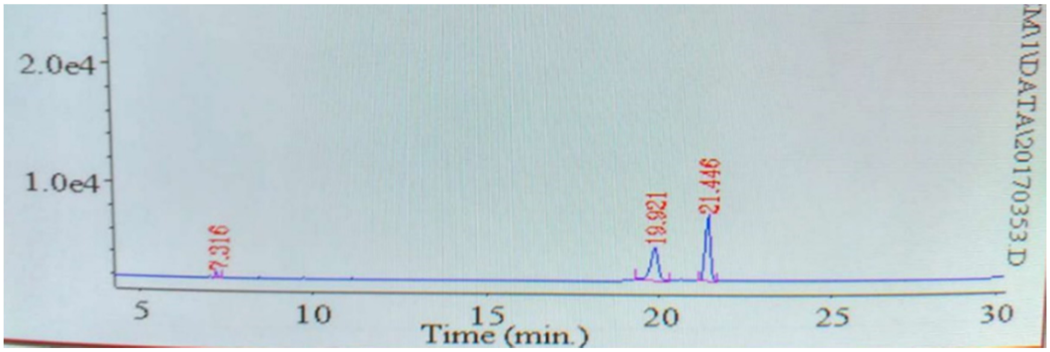

Figure S6. GC analysis of the derivative of compound **2** after hydrolysis

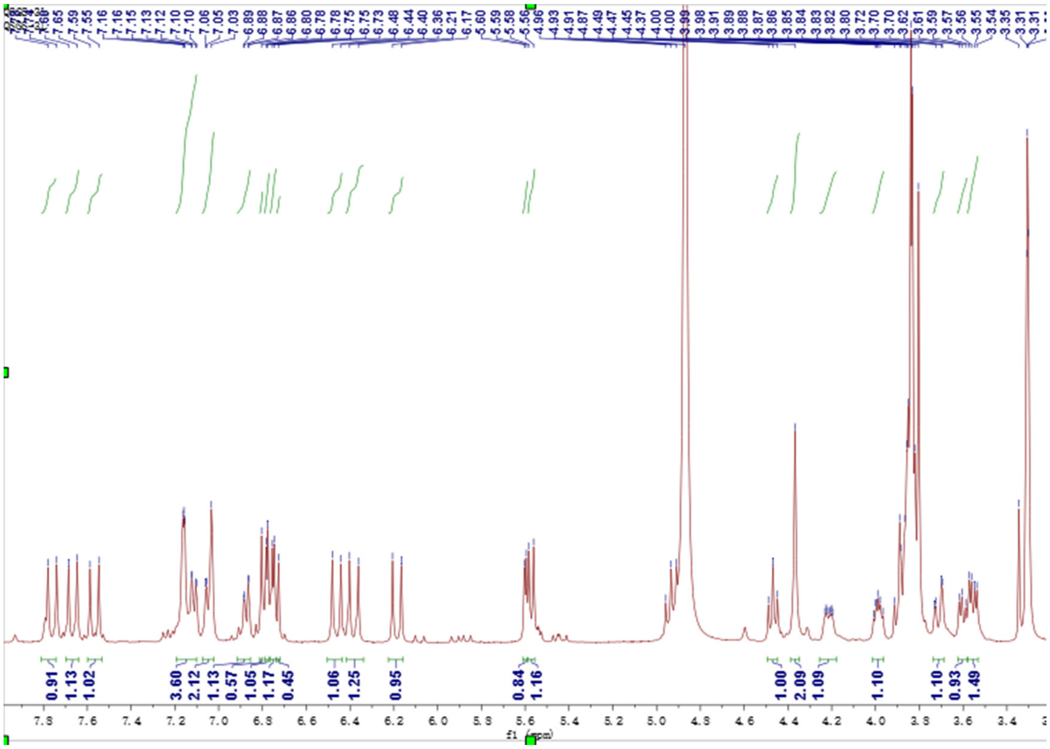

Figure S7. The ^{1}H NMR spectrum of **1** in CD_3OD

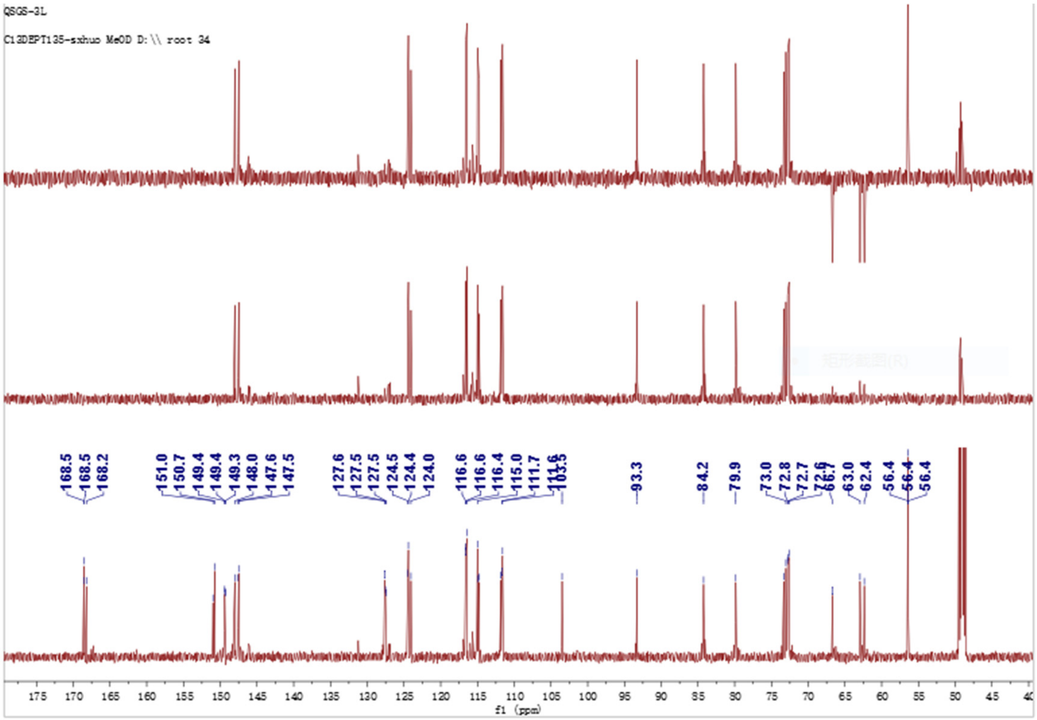

Figure S8. The ^{13}C NMR and DEPT spectra of **1** in CD_3OD

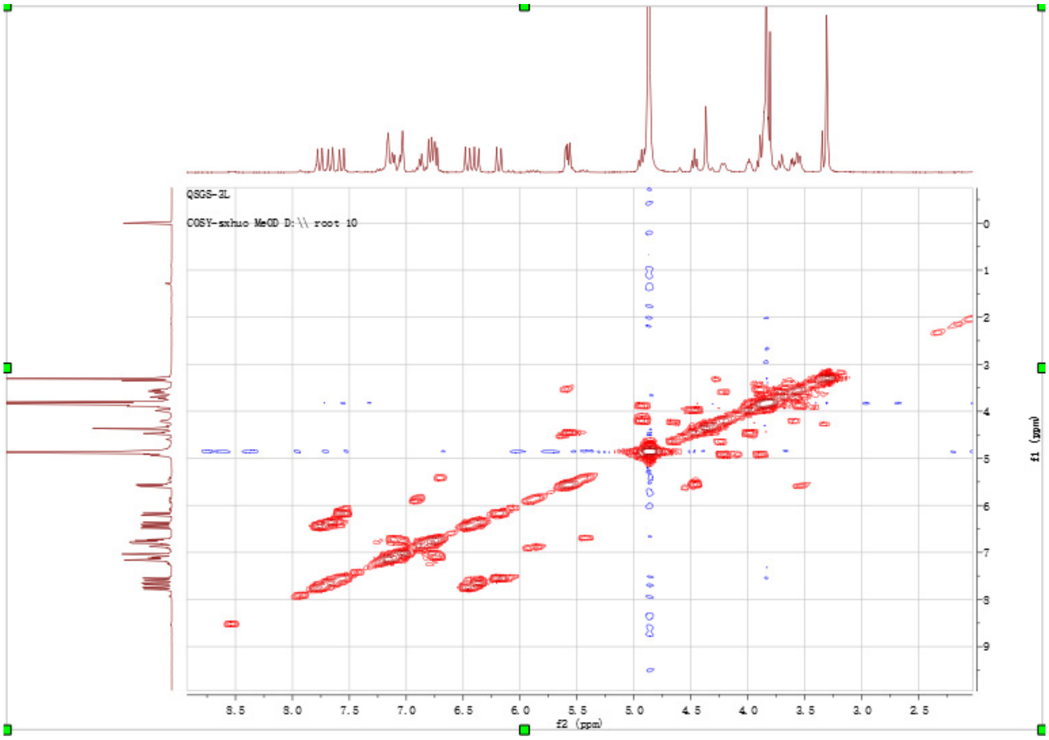

Figure S9. ^1H-^1H COSY spectrum of **1** in CD_3OD

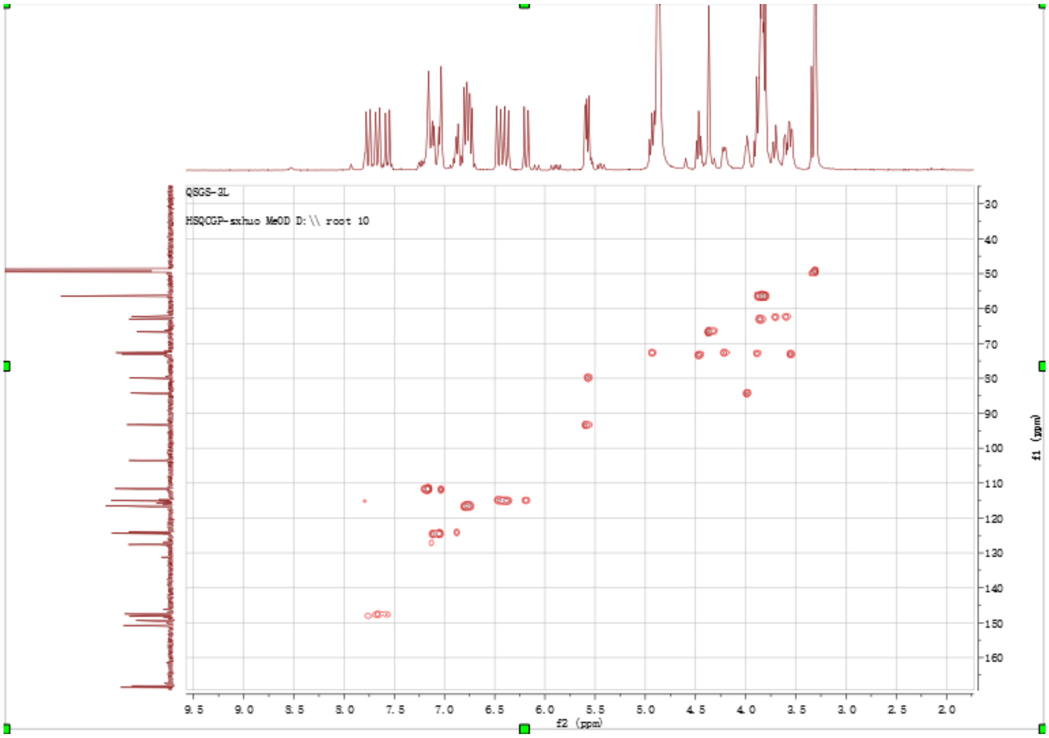

Figure S10. HSQC spectrum of **1** in CD_3OD

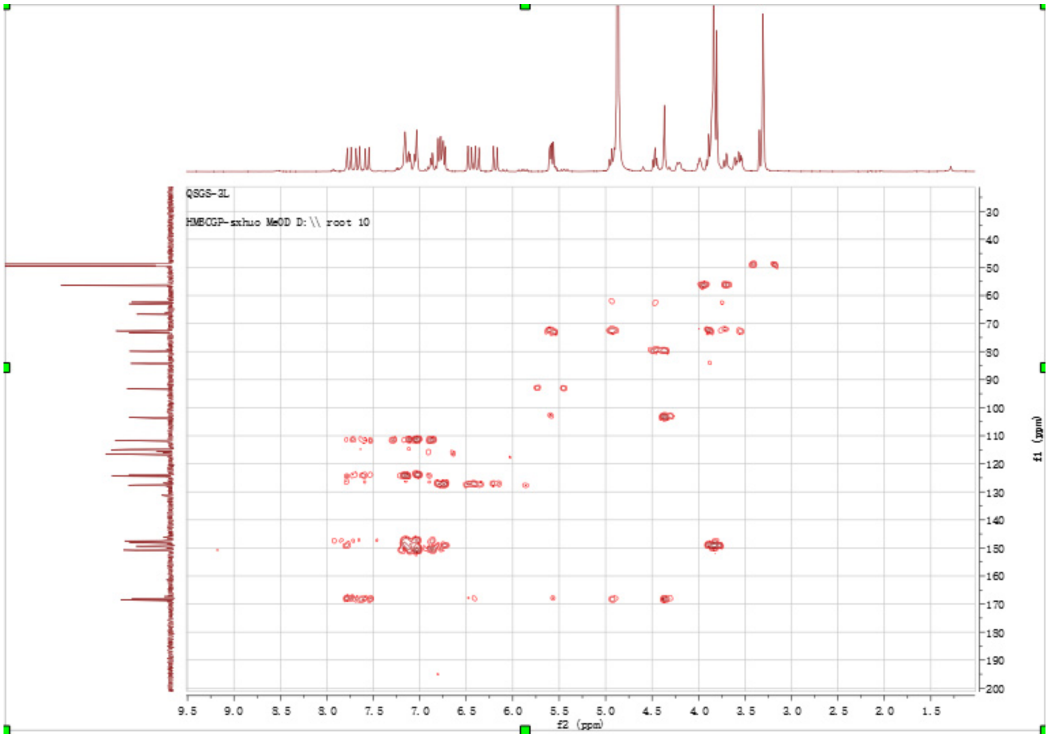

Figure S11. HMBC spectrum of **1** in CD_3OD

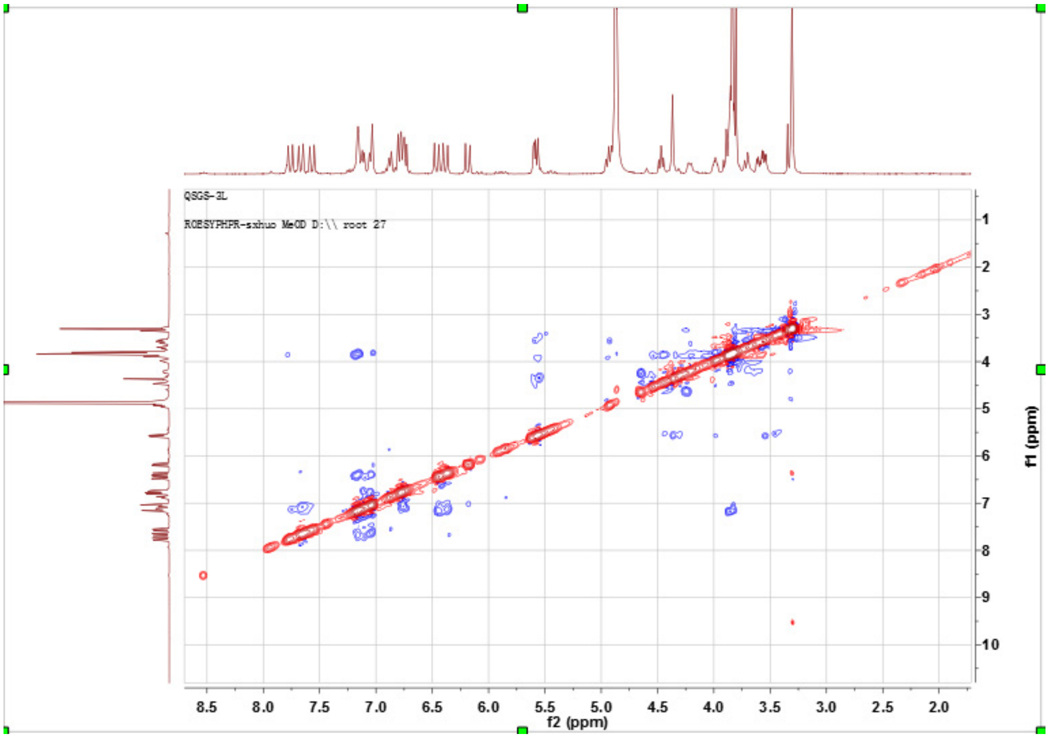

Figure S12. ROESY spectrum of **1** in CD_3OD

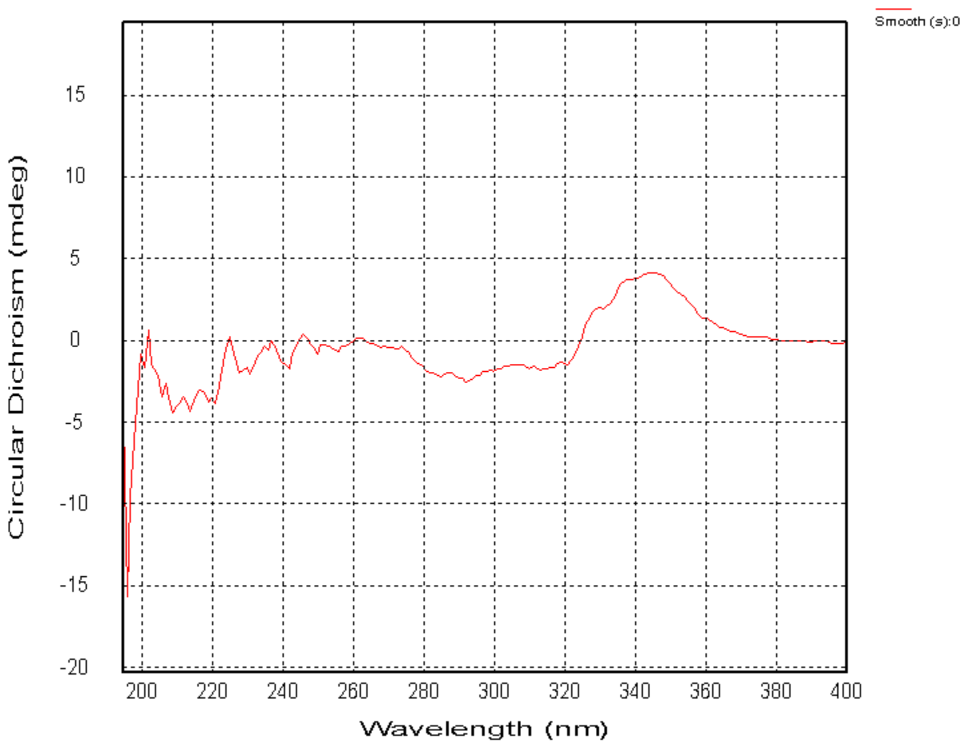

Figure S13. CD spectrum of **1**

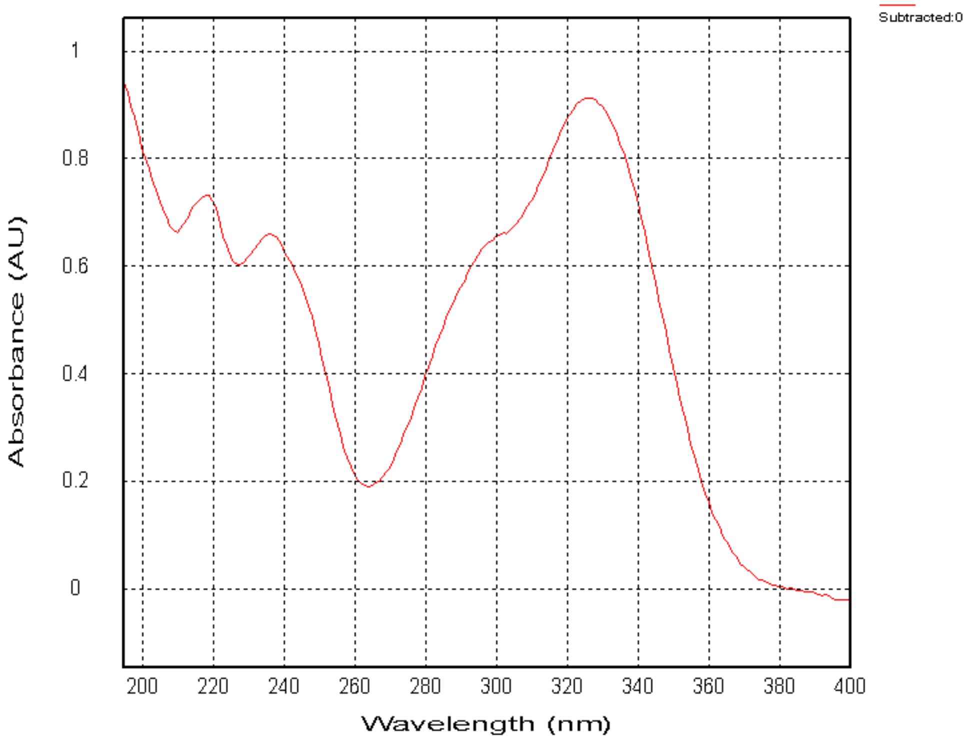

Figure S14. UV spectrum of **1**

Formula Predictor Report - QSGS-3L.lcd

Page 1 of 1

Data File: E:\DATA\2017\0927\QSGS-3L.lcd

Elmt	Val.	Min	Max	Elmt	Val.	Min	Max	Elmt	Val.	Min	Max	Elmt	Val.	Min	Max	Use Adduct
H	1	0	100	O	2	0	50	P	3	0	0	Br	1	0	0	Na
B	3	0	0	F	1	0	0	S	2	0	5	I	3	0	0	
C	4	0	100	Na	1	0	0	Cl	1	0	0	Pt	2	0	0	
N	3	0	0	Mg	2	0	0	Fe	2	0	0					

Error Margin (ppm): 5
HC Ratio: unlimited
Max Isotopes: all
MSn Iso RI (%): 75.00

DBE Range: -2.0 - 100.0
Apply N Rule: yes
Isotope RI (%): 1.00
MSn Logic Mode: AND

Electron Ions: both
Use MSn Info: yes
Isotope Res: 10000
Max Results: 10

Event#: 1 MS(E+) Ret. Time : 0.390 -> 0.470 Scan# : 79 -> 95

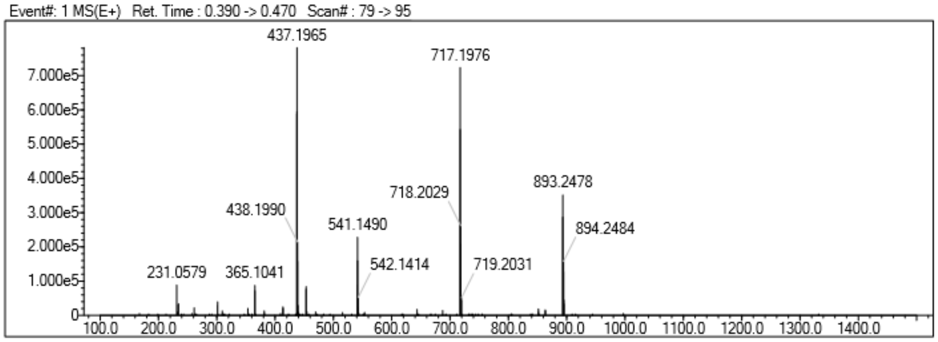

Measured region for 893.2478 m/z

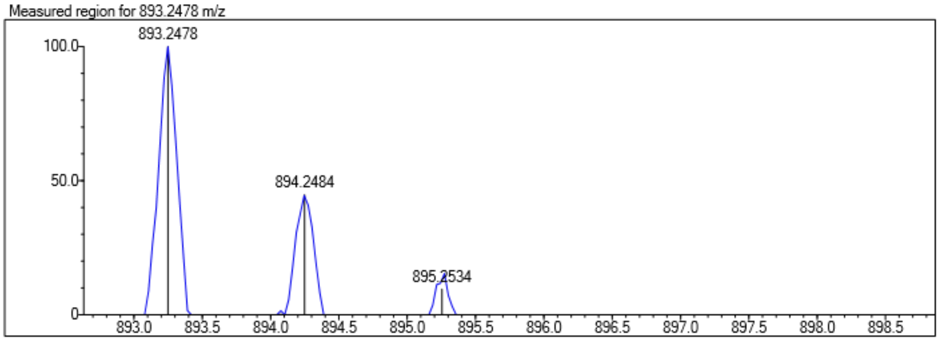

C42 H46 O20 [M+Na]+ : Predicted region for 893.2475 m/z

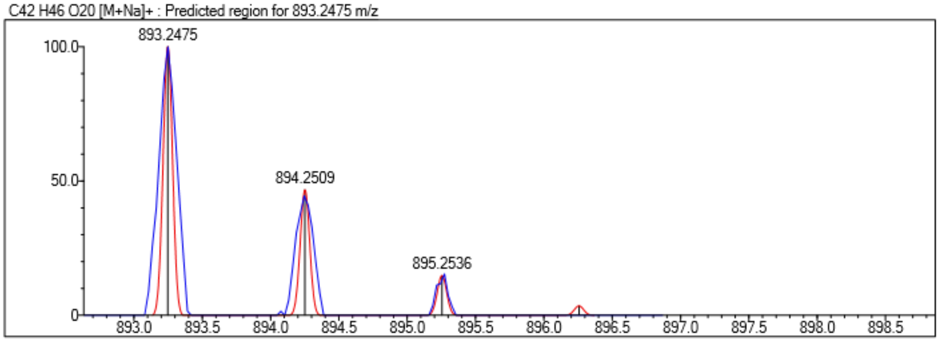

Formula (M)	Ion	Meas. m/z	Pred. m/z	Df. (mDa)	Df. (ppm)	DBE
C42 H46 O20	[M+Na]+	893.2478	893.2475	0.3	0.34	20.0

Figure S15. HRESIMS of **1**

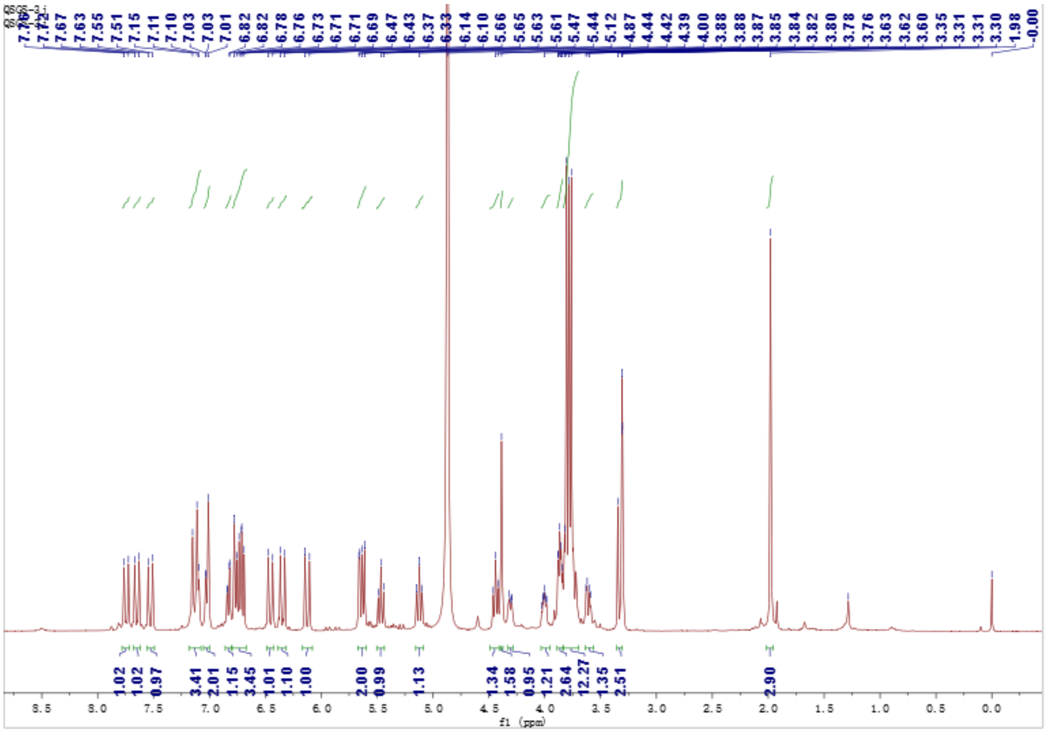

Figure S16. The ^{1}H NMR spectrum of **2** in CD_3OD

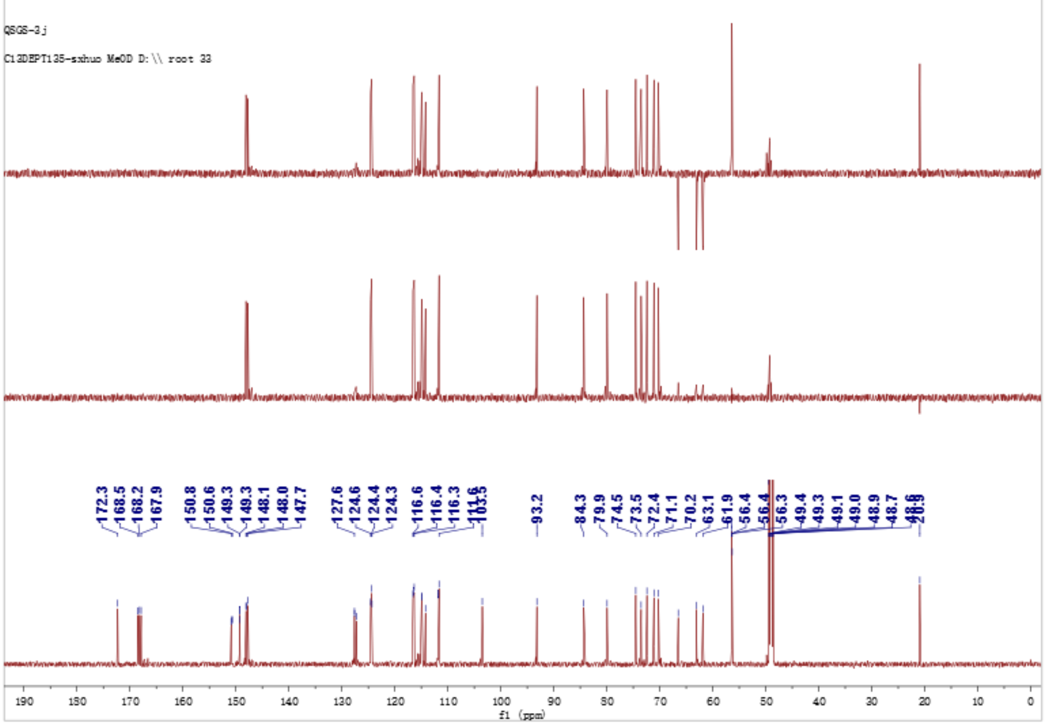

Figure S17. The ^{13}C NMR and DEPT spectra of **2** in CD_3OD

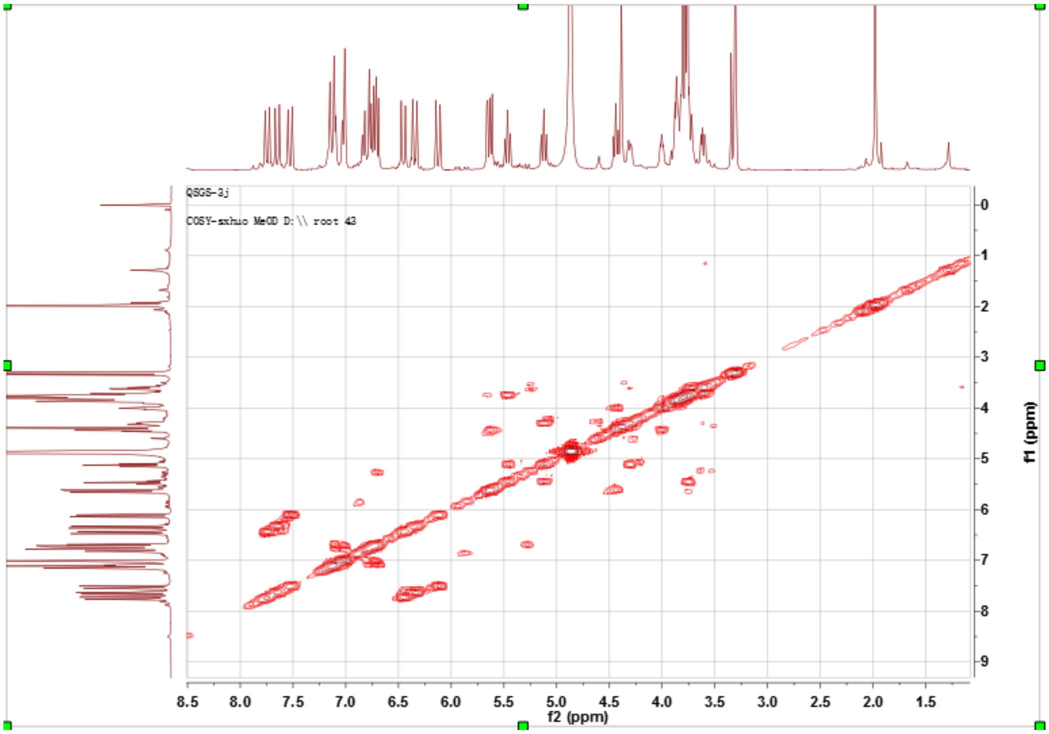

Figure S18. ^{1}H-^{1}H COSY spectrum of **2**

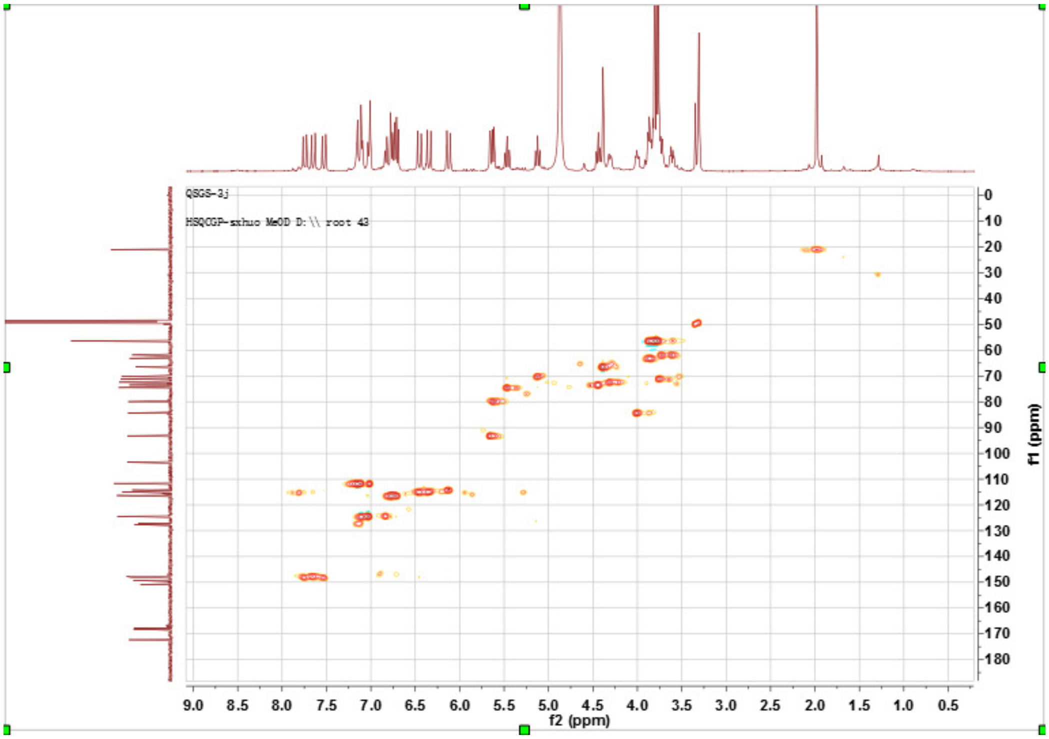

Figure S19. HSQC spectrum of **2**

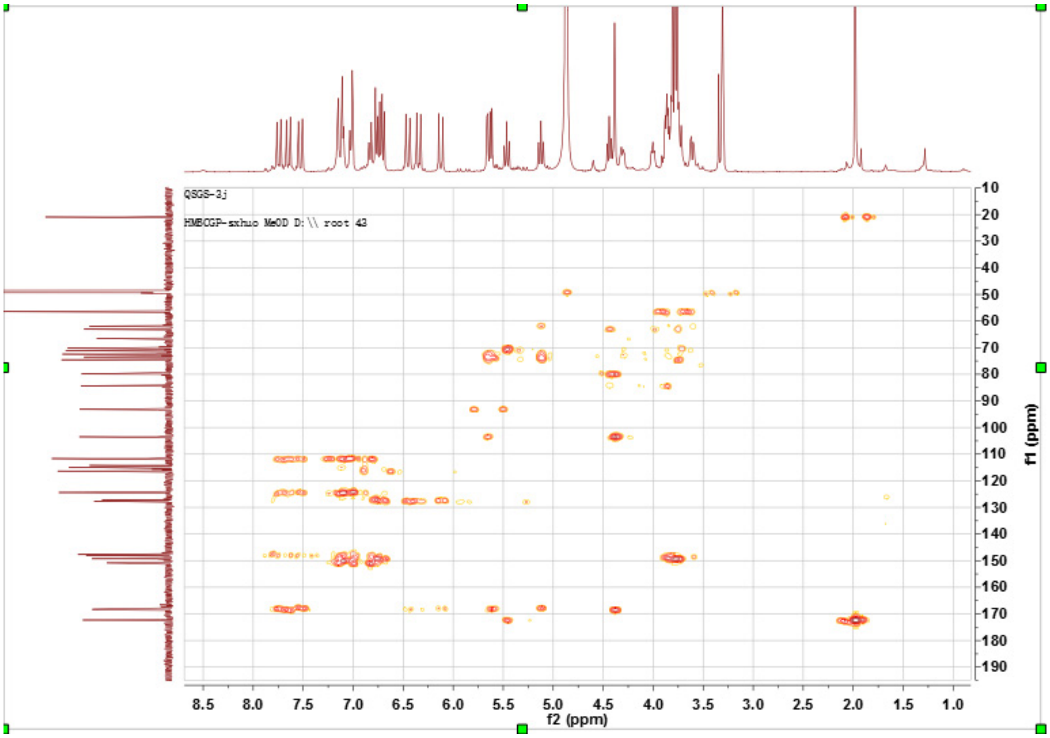

Figure S20. HMBC spectrum of **2**

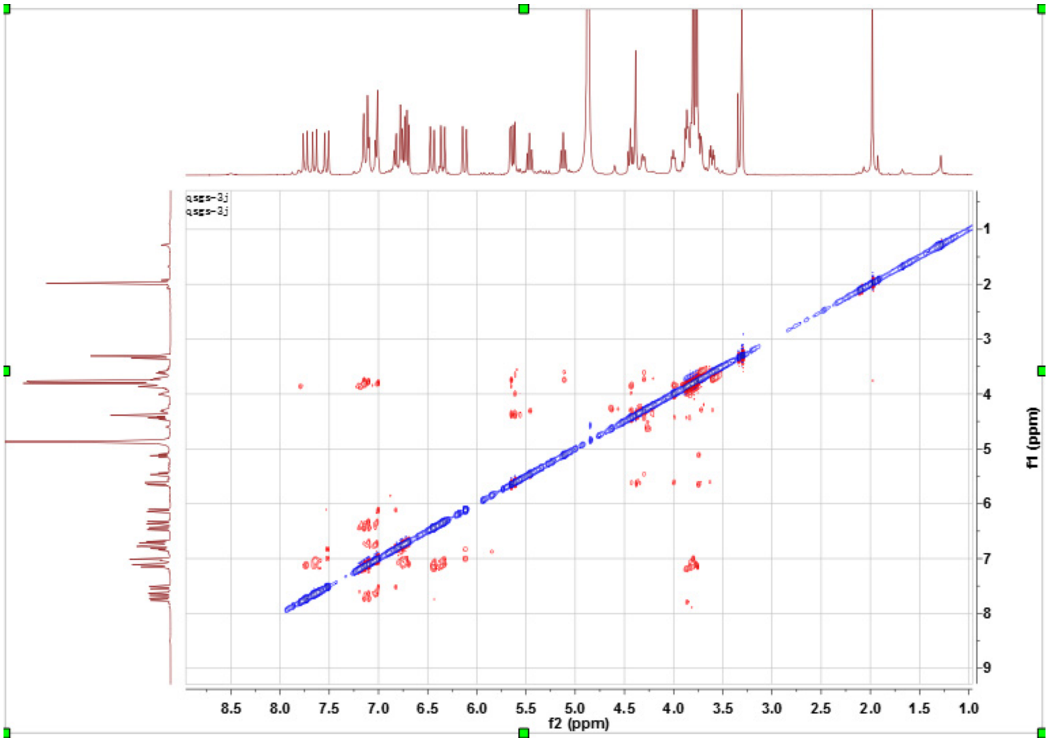

Figure S21. ROESY spectrum of **2**

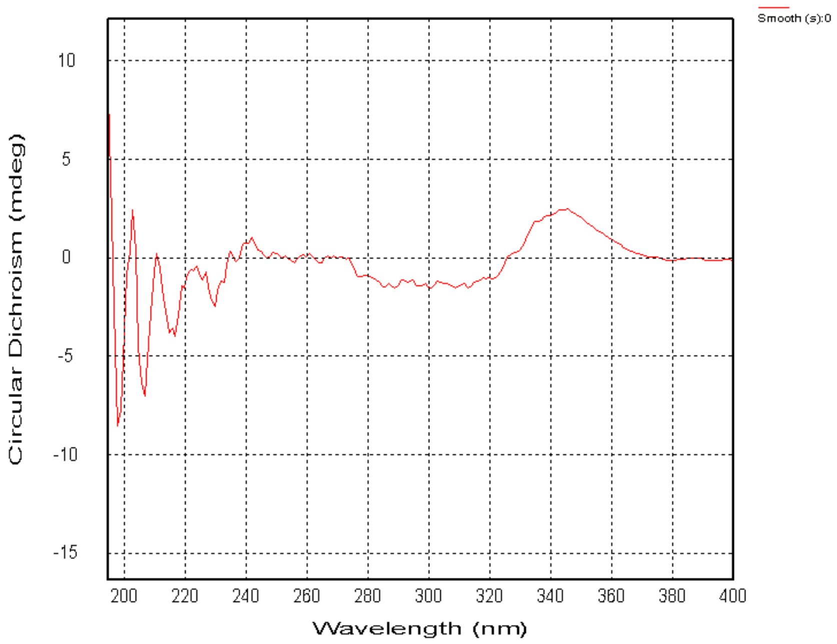

Figure S22. CD spectrum of **2**

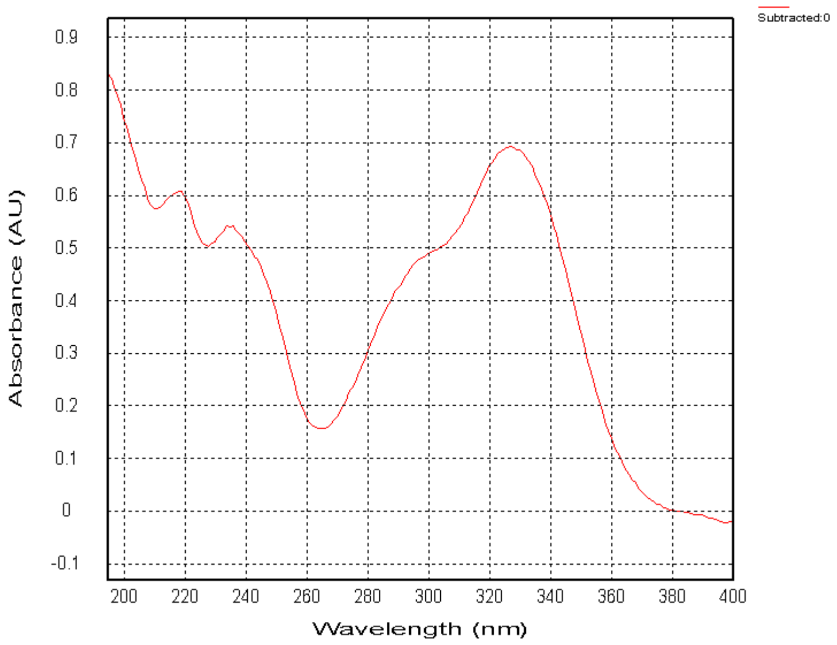

Figure S23. UV spectrum of **2**

Formula Predictor Report - QSGS-3j.lcd Page 1 of 1

Data File: E:\DATA\2017\0927\QSGS-3j.lcd

Elmt	Val.	Min	Max	Elmt	Val.	Min	Max	Elmt	Val.	Min	Max	Elmt	Val.	Min	Max	Use Adduct
H	1	0	100	O	2	0	50	P	3	0	0	Br	1	0	0	Na
B	3	0	0	F	1	0	0	S	2	0	5	I	3	0	0	
C	4	0	100	Na	1	0	0	Cl	1	0	0	Pt	2	0	0	
N	3	0	0	Mg	2	0	0	Fe	2	0	0					

Error Margin (ppm): 5
HC Ratio: unlimited
Max Isotopes: all
MSn Iso RI (%): 75.00

DBE Range: -2.0 - 100.0
Apply N Rule: yes
Isotope RI (%): 1.00
MSn Logic Mode: AND

Electron Ions: both
Use MSn Info: yes
Isotope Res: 10000
Max Results: 10

Event#: 1 MS(E+) Ret. Time : 0.320 -> 0.480 Scan# : 65 -> 97

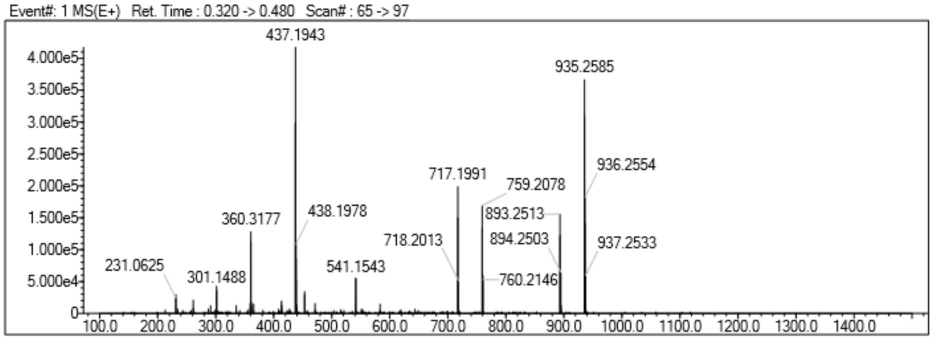

Measured region for 935.2585 m/z

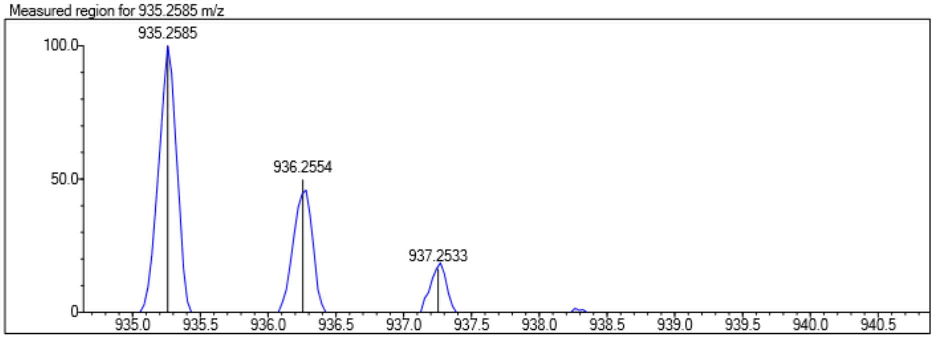

C44 H48 O21 [M+Na]+ : Predicted region for 935.2580 m/z

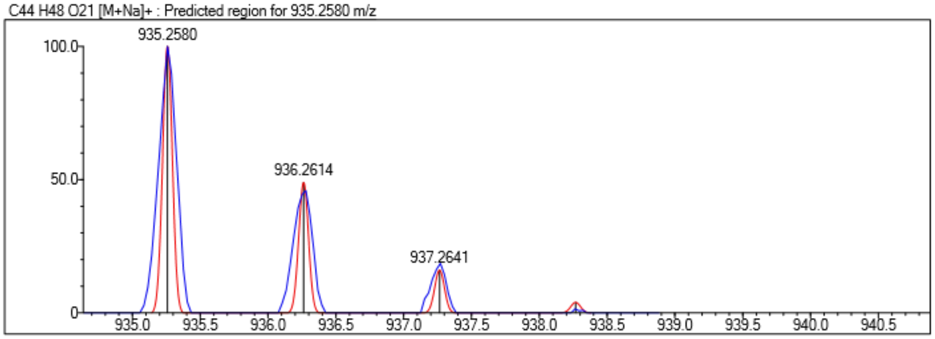

Formula (M)	Ion	Meas. m/z	Pred. m/z	Df. (mDa)	Df. (ppm)	DBE
C44 H48 O21	[M+Na]+	935.2585	935.2580	0.5	0.53	21.0

Figure S24. HRESIMS of **2**
